# Bone mineral density reference values in Singaporean adults and comparisons for osteoporosis establishment – The Yishun Study

**DOI:** 10.1186/s12891-020-03646-y

**Published:** 2020-09-25

**Authors:** Kexun Kenneth Chen, Shiou-Liang Wee, Benedict Wei Jun Pang, Lay Khoon Lau, Khalid Abdul Jabbar, Wei Ting Seah, Sivasubramanian Srinivasan, Mallya Ullal Jagadish, Tze Pin Ng

**Affiliations:** 1 Geriatric Education and Research Institute (GERI), 2 Yishun Central 2, Tower E Level 4 GERI Admin, Singapore, 768024 Singapore; 2 Health and Social Sciences Cluster, Singapore Institute of Technology, Singapore, Singapore; 3 Programme of Health Services and System Research, Duke-National University of Singapore Graduate Medical School, Singapore, Singapore; 4 Diagnostic Radiology, Khoo Teck Puat Hospital, Singapore, Singapore; 5 Geriatric Medicine, Khoo Teck Puat Hospital, Singapore, Singapore; 6 Department of Psychological Medicine, National University of Singapore, Singapore, Singapore

**Keywords:** Bone mineral density, Reference range, Osteoporosis, Prevalence, Normative

## Abstract

**Background:**

While there have been studies in Singapore on the prevalence and economic burden of osteoporotic hip fracture, there is a severe lack of reference data on bone mineral density and prevalence of osteoporosis. The purpose of this study is to establish the reference values for BMD and compare prevalence of osteoporotic conditions using other available reference values so as to better understand the status of bone health in Singaporean adults.

**Methods:**

We carried out a population-based cross-sectional study using dual-energy x-ray absorptiometry (Hologic Discovery Wi) to measure the bone mineral density of Singaporean adults aged ≥21 years. A total of 542 participants were recruited from the large north-eastern residential town of Yishun. ﻿We computed T- scores (denoted by T_SG_) for each individual in the study. Similar diagnoses were also done based on T-scores provided by the densitometer (T_DXA_), NHANES database (T_NHANES_), and China (T_CHN_), and the differences in prevalence compared. We then compared the concordance between T_SG_ and T_DXA_ in the classification of osteoporosis. Osteoporosis was defined according to criteria by the World Health Organization (WHO).

**Results:**

Peak lumbar spine BMD was 1.093 ± 0.168 g/cm^2^ in women, and 1.041 ± 0.098 g/cm^2^ for men. Peak whole-body BMD was 1.193 ± 0.93 g/cm^2^ in women at, and 1.224 ± 0.112 g/cm^2^ for men. Prevalence of osteoporosis based on lumbar spine was 9.3% in postmenopausal women, and 0.7% in men after 50 years of age. The percentage difference in prevalence range from 60.5–163.6%, when using reference values from T_DXA_, T_NHANES_, and T_CHN_. Comparing diagnosis using T_DXA_ and T_SG_ cut-off values, 28 versus 15 women were diagnosed as osteoporotic respectively. ﻿ The kappa statistics was 0.81 for women and 0.85 for men.

Conclusion: ﻿Our study shows that T-scores provided by DXA manufacturer over-diagnosed osteoporosis in Singaporeans, and the prevalence of osteoporotic conditions is not accurately represented. This over-diagnosis may result in unnecessary treatment in some individuals.

## Introduction

Osteoporosis is characterised by a systemic loss of bone mineral and micro-architectural deterioration of bone tissue, resulting in an increased risk of fracture [1]. Osteoporotic fractures result in increased morbidity, disability, and mortality risk, reducing the quality of life [2]. Common fracture sites include the spine, hip, distal forearm, and proximal humerus. The average risk of osteoporotic fracture for people at 50 years of age has been estimated to be 40% for women, and 13% for men [3]. Osteoporosis and associated fractures have become increasingly common in ageing populations and is a public health issue [1].

Measurement of bone mineral density (BMD) using dual-energy X-ray absorptiometry (DXA) is the most widely used indicator of bone health and for detection of osteoporosis [4]. Most studies to accumulate BMD reference data are from America or Europe with relatively few Asian studies. Manufacturer-supplied BMD reference values from Western populations may not be fully representative of Asian populations and can, therefore, lead to estimates of the prevalence of osteoporosis that differ greatly from those calculated based on a local reference population [5].

A multi-ethnic country in southeast Asia, Singapore is ageing rapidly with the proportion of resident population aged 65 years and over having risen from 9.0% in 2010 to 14.4% in 2019 [6], and is expected to reach 25% by 2030 [7]. Osteoporotic hip fracture incidence rates have risen 1.5-fold for men and 5-fold for women since the 1960s [8]. Between 2000 to 2017, there is a 3.5% increase in hip-fracture related hospitalization, translating to an average of 72 additional hip fractures related hospitalisation per 100,000 per year [9]. In 2017, the estimated incidence of osteoporotic fracture was 15,267 case, (25.5% hip fracture, 29.1% vertebral fracture, and 45.4% other fractures), and it is estimated to increase by 58% by 2035 [10]. The total economic burden associated with osteoporosis and related fractures was estimated to be S$183.5 million in 2017, and forecast to increase to S$289.6 million by 2035 [10]. Mortality rate one year post osteoporotic hip fracture was reported to be 20–27%, with most survivors became semi or fully dependent (20%), or experienced reduced mobility status (39–42%) [8, 11]. Only 26% were cared for by chronic health care facilities suggesting that the main social and financial burden was borne by the family caregivers [8, 11, 12].

While there have been studies in Singapore on the prevalence and economic burden of osteoporotic hip fracture [8, 9, 11], there is a severe lack of reference data on BMD and prevalence of osteoporosis. A study published in 2002, found Singapore men had 10 and 5% lower bone mineral density in the lumbar spine (LS) and femoral neck, respectively when compared to mean BMD peak of Caucasian reference database [8]. A previous study on women aged 20–59 years reported no difference in peak BMD in the LS and femoral neck between the three main ethnic groups of Singapore [13]. Previous studies also reported that Chinese women have the lowest femoral neck BMD compared to Malay and Indians [13]. However, there is no study on the prevalence of osteoporotic conditions or women BMD reference values in Singapore.

World Health Organisation (WHO) defined osteoporosis as having BMD of 2.5 standard deviations (SD) or more below the young female adult mean, using normative data from NHANES reference database on Caucasian women aged 20–29 [2]. However, peak BMD differs among ethnic and gender groups [14–16]. The cut-off values for defining osteoporosis specific to Asian populations are only available for Taiwan [17], South Korea [18], China [19, 20], Japan [21, 22], and Vietnam [15]. Singapore’s multi-ethnic population comprised 74.4% Chinese, 13.4% Malay, 9.0% Indians, and 3.2% of other races [6].

The purpose of this study is to establish BMD reference values for Singapore and compare the prevalence of osteoporosis by using our BMD data with those calculated from other available reference values. We hypothesise that there are considerable differences in the diagnosis of osteoporosis between DXA-machine provided reference data from other populations with our locally derived reference data.

## Methods

### Settings

The study was designed as a cross-sectional investigation. Community-dwelling adults (≥21 years) were recruited from a large north-eastern residential town of Yishun Singapore, with residential population of 220,320 (49.4% men), with 12.2% older adults (≥65 years) [6]. This is similar to the overall Singapore residential population of 4.02 million (48.9% men), with 14.4.% older adults (≥65 years) [6].

### Study design and participants

A total of 542 participants were recruited for this study. A priori random sampling methodology was employed to obtain a representative sample of approximately 300 male and 300 female participants, filling quotas of 20–40 participants in each sex- and age-group (10-year age-groups between 21 and 60 years; 5-year age-groups after 60 years). Conventionally, the sample size of 30 per age-group is sufficient for normative measures [23]. Between October 2017 and February 2019, using a two-stage random sampling method, 50% of all housing blocks were randomly selected, and a random 20% of the units in each block were approached for participant recruitment. Between March and November 2019, 50% of all housing blocks were randomly selected and all units were approached. Up to three eligible participants were recruited from each housing unit using a door-to-door recruitment method. Non-response units were re-contacted a second time at a different time of day on a later date. Older adults above 75 years old were additionally recruited through community sources and from a list of registered participants in four senior activity centres. Exclusion criteria were: individuals with disabilities, injuries, fractures or surgeries that affected function, neuromuscular, neurological and cognitive impairments, or more than five poorly controlled comorbidities. Pregnant women or those planning for pregnancy were also excluded. The estimated overall response rate was 39.0%. Ethics approval was obtained from the National Healthcare Group Domain Specific Review Board (2017/00212). All respondents signed informed consent before their participation in the study.

### Measurements and data collection

Data collection was conducted by research officers using validated questionnaires. Participants answered a health and medical questionnaire indicating history of medical conditions and comorbidities. Menopausal status and hysterectomy were based on self-report.

### Anthropometry

Body weight to the nearest 0.1 kg and height to nearest millimetre were measured using a digital balance and stadiometer (Seca, GmbH & Co. KG, Hamburg, Germany). Height measurement was then converted to the centimetre (cm). Waist and hip circumferences were measured to the nearest centimetre, using a non-elastic, flexible measuring tape around the navel and widest part of the hips respectively. Body mass index (BMI) was calculated as weight (kg) divided by height (m) squared.

### Bone mineral density

Areal BMD was measured for the whole-body using Hologic Discovery Wi (Hologic, Marlborough, MA, USA) in a supine position. The densitometry scan was conducted by experienced radiographers. Longitudinal quality control (QC) check and cross-calibrations were performed regularly. QC was performed daily using whole-body and L1-L4 lumbar spine phantom provided by the manufacturer. Cross-calibration was performed weekly to monitor variations between the systems. Air scan test was performed weekly for table top uniformity. All participants were provided with a standard hospital gown, and requested to remove all garments and objects with at would potentially interfere with DXA scan. Lumbar spine BMD was extracted from the whole-body scan. Osteoporosis cut-off value was based on WHO diagnostic criteria using ﻿SD scores of BMD related to peak bone mass in healthy young women. Osteoporosis is defined as having a BMD T-score of − 2.5 or less, and osteopenia being defined as a BMD T-score of between − 1 and − 2.5. Using young adult mean (YAM) and SD derived from this study and other studies [20, 24], T-scores were calculated for postmenopausal women, and men aged 50 and above. T-score derived using YAM from our study is referred to as T_SG_, T_DXA_ refers to T-score calculated using Hologic densitometry reference value, T_NHANES_ were derived from using NHANES database [24], and T_CHN_ is from China cut-off values [20].

### Statistical analysis

All statistical analyses were performed using SPSS Statistics version 22.0 (IBM, Armonk, NY, USA). Normality of the variables was examined using histogram. The sex differences for each continuous variable were calculated using the independent t-test. Relationship between BMD and age were analysed using polynomial regression model (up to the third degree). Models were fitted to the LS and WB BMD as a function of age as shown: BMD = α + β_1_(age) + β_2_(age)^2^ + β_3_(age)^3^, where α is the intercept, and β_1_, β_2_, β_3_ are regression parameters, which were estimated from observed data. Linear and quadratic models were fitted, and the best-fit model was chosen based on the R^2^ value, and the significance R^2^ changes between each degree model. Statistical agreement between Singapore cut-off value and DXA manufacturer reference value was analysed using kappa statistics. In post-hoc analysis, LS BMD differences between age groups in men was analysed using ANOVA test and Turkey’s honestly significant difference (HSD). All data are presented in mean ± standard deviation, unless otherwise stated.

## Results

Due to difficulty in recruiting participants in the older age group, a total of 542 subjects were recruited for the study. Hence, resulting in 9 age categories, and a smaller study sample of 16 to 31 subjects per category (Table 2). Four subjects withdraw from the study, and one subject did not undergo DXA scan due to possibility of pregnancy. A total of 537 subjects (58.5% women) aged 21 and above, received the DXA scan. A total of 72 subjects were excluded due to poor image scan (i.e. having joint replacement surgery, metal implants, or unable to have a full body DXA scan). Of the remaining 465 participants (55.9% women), 34.8% of women were postmenopausal and 30.7% of men were above the age of 50. Ethnic composition is 81% Chinese, 9% Malay, 6% Indians, and 4% others - similar to national population [6]. Women’ mean weight was 58.1 ± 10.0 kg, mean height was 155.4 ± 6.3 cm, mean BMI was 24.1 ± 3.9, and the average age of menopause is 51 years. Men were heavier and taller - mean weight was 68.5 ± 11.9 kg, 166.5 ± 6.75 cm, and mean BMI was 24.6 ± 3.7 (Table 1).

**Table 1:** Participants demographics

	Women (***n*** = 260)	Men (***n*** = 205)	***P***-value
**Age (years)**	56.7 ± 18.3	59.4 ± 18.8	0.120
**Weight (kg)**	58.1 ± 10.0	68.5 ± 11.9	< 0.001
**Height (cm)**	155.4 ± 6.3	166.5 ± 6.75	< 0.001
**BMI (kg.m**^**−2**^**)**	24.1 ± 3.9	24.6 ± 3.7	0.124
**Race (%)**			
Chinese	81.15	82.44	
Malay	10.39	7.32	
Indian	5.77	7.32	
Others	2.69	2.92	

Data presented in mean ± standard deviation, unless otherwise stated

*BMI* body mass index

### Normative bone mineral density values

**Fig. 1:**
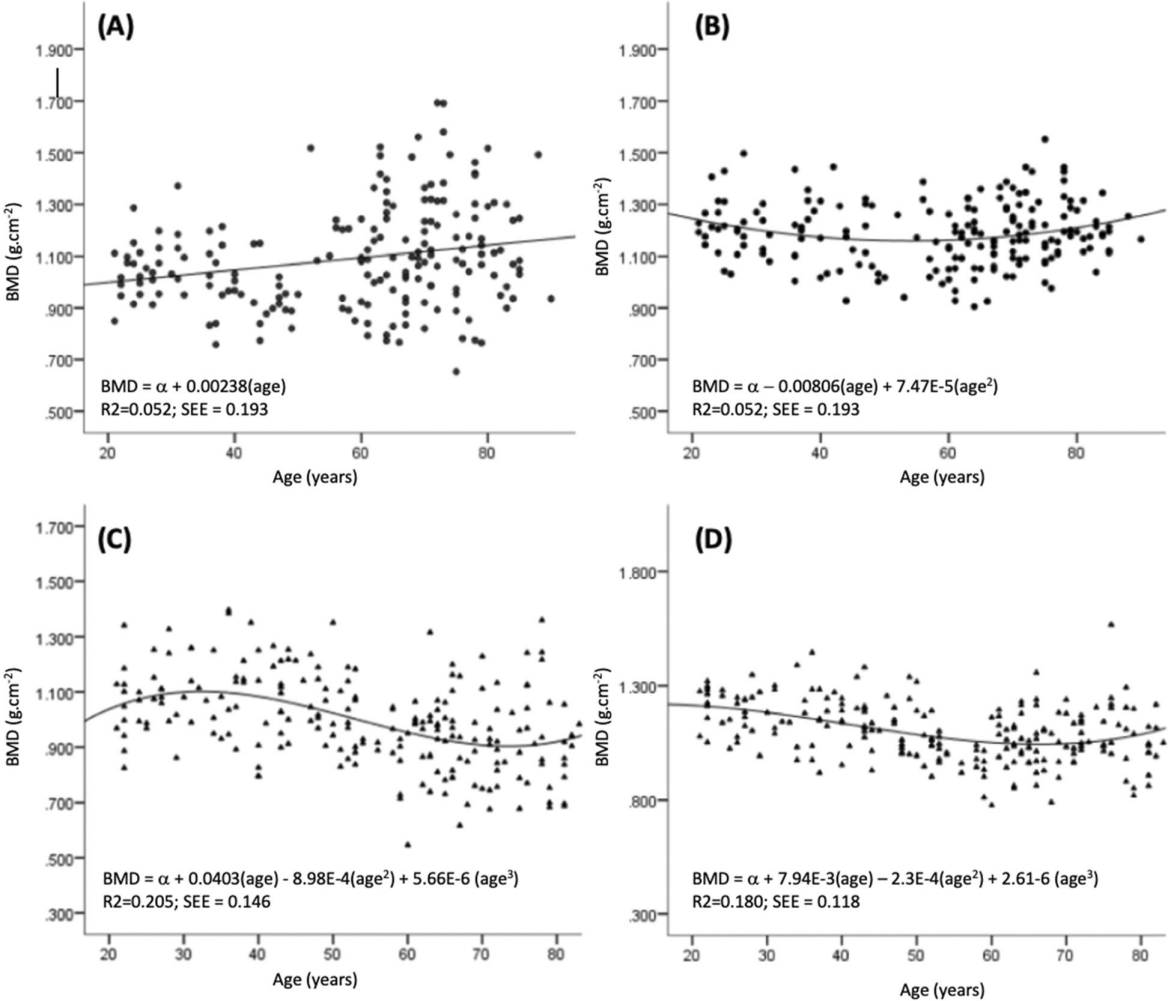
Relationship between age and bone mineral density: (**a**) Men, Lumbar spine; (**b**) Men Whole body; (**c**) Women, Lumbar spine; (**d**) Women, Whole body

Table 2 shows height, weight, BMI, waist and hip circumference of participants. To calculate the normative BMD, a further 53 subjects with existing medical conditions (i.e. endocrine disorders, liver or renal disease, diabetes, or rheumatoid arthritis), and/or on medication or supplements (i.e. calcium supplements, fluorides, steroids, or oestrogen) that may have an effect on bone metabolism or BMD, were excluded from the analysis. Data from 413 subjects (223 women and 190 men) were used to compute normative BMD values. BMD, whole-body (WB) and posterior anterior lumbar spine (LS) BMD of different age groups are presented in Table 3. Peak LS mean BMD was 1.090 ± 0.168 g/cm^2^ in the 31–40 age group for pre-menopausal women, and 1.041 ± 0.098 g/cm^2^ in 21–30 age group for men < 50 yrs. of age. Peak WB mean BMD was 1.193 ± 0.93 g/cm^2^ in 21–30 age group for pre-menopause women at, and 1.224 ± 0.112 g/cm^2^ in the 21–30 age group for men < 50 yrs. of age. A cubic polynomial regression model had best fit relationship between BMD and age for women (Fig. 1**c**). The relationship between BMD and age in women, can be identified in three stages, a gradual increase between ages of 20 and 30, followed by a steady period between ages of 30 and 40, then a gradual decline after 40 years of age. In men, linear and quadratic regression curve (Fig. 1a, b) best described the relationship of LS and WB BMD with age respectively. Men’s LS BMD seems to increase with age. WB BMD peaks before 20 years of age, followed by a gradual decline between age of 20–40, a steady period between 40 to 60 years of age, then a gradual incline after 60 years of age. However, post-hoc analysis indicated insignificant difference in LS and WB BMD across age group.

**Table 2:** Participants height, weight, BMI, waist and hip circumference

	Women						Men					
Age (yr)	n	Height (cm)	Weight (kg)	BMI (kg.m^**−2**^)	Waist Circumference (cm)	Hip Circumference (cm)	n	Height (cm)	Weight (kg)	BMI (kg.m^**−2**^)	Waist Circumference (cm)	Hip Circumference (cm)
**Total**	223	155.6 ± 6.5	59.0 ± 10.1	24.4 ± 4.0	85.1 ± 10.8	98.5 ± 7.7	190	166.7 ± 6.8	68.8 ± 12.0	24.7 ± 3.7	90.2 ± 9.5	97.1 ± 6.8
**21–30**	31	160.3 ± 5.5	57.2 ± 11.5	22.3 ± 4.5	76.8 ± 11.8	95.5 ± 8.2	24	172.9 ± 5.4	72.9 ± 12.0	24.4 ± 3.8	85.2 ± 10.6	97.8 ± 6.8
**31–40**	29	158.5 ± 5.4	60.4 ± 10.3	24.1 ± 4.0	82.1 ± 9.1	98.0 ± 8.0	21	168.9 ± 4.2	75.3 ± 13.9	26.3 ± 4.4	89.5 ± 11.8	99.7 ± 8.8
**41–50**	32	157.6 ± 6.8	63.3 ± 10.8	25.5 ± 4.0	84.5 ± 11.2	99.7 ± 7.8	18	167.3 ± 6.1	77.3 ± 14.0	27.5 ± 3.9	94.8 ± 8.6	99.4 ± 7.9
**51–60**	30	156.6 ± 6.4	62.1 ± 12.4	25.3 ± 4.7	86.8 ± 12.1	100.4 ± 9.1	16	169.8 ± 7.2	73.9 ± 9.2	25.7 ± 2.9	92.2 ± 7.9	98.4 ± 6.5
**61–65**	28	155.3 ± 4.9	58.5 ± 8.0	24.3 ± 3.5	86.7 ± 9.2	98.5 ± 7.1	27	166.4 ± 5.9	66.5 ± 8.1	24.0 ± 2.9	89.8 ± 8.0	95.6 ± 4.7
**66–70**	22	152.6 ± 4.9	58.3 ± 6.0	25.1 ± 2.7	89.6 ± 7.4	100.0 ± 7.0	24	165.6 ± 4.8	66.5 ± 10.5	24.2 ± 3.4	90.4 ± 8.9	96.6 ± 6.7
**71–75**	18	152.9 ± 5.4	52.4 ± 6.4	22.5 ± 3.1	86.8 ± 10.1	95.6 ± 3.9	24	164.1 ± 6.1	64.2 ± 8.6	23.9 ± 3.4	91.0 ± 9.9	95.8 ± 5.6
**76–80**	17	150.6 ± 4.5	58.9 ± 9.4	26.0 ± 3.8	90.2 ± 9.1	99.6 ± 7.2	19	163.1 ± 7.6	62.1 ± 8.9	23.3 ± 2.5	89.5 ± 8.9	95.0 ± 6.3
**81+**	16	147.9 ± 4.6	54.1 ± 7.8	24.8 ± 3.8	89.1 ± 8.3	99.5 ± 8.2	17	161.7 ± 7.3	62.1 ± 12.4	23.7 ± 4.1	91.3 ± 9.5	96.7 ± 7.4

Data presented in mean ± standard deviation. n: actual number of individuals in each subgroup

**Table 3:** BMD in different age groups

	Women				Men			
Age group	Singapore	Goh et al.	NHANES	China	Singapore	Thoo et al.	NHANES	China
	**Lumbar Spine**							
21–30	1.071 ± 0.121	1.035^d^	1.064 ± 0.106^a^	1.061 ± 0.124^c^	1.041 ± 0.098	1.002 ± 0.101^e^	1.057 ± 0.110^a^	1.073 ± 0.138^c^
31–40	1.090 ± 0.168	1.053	1.065 ± 0.110	1.090 ± 0.126	1.040 ± 0.143	0.989 ± 0.117	1.042 ± 0.117	1.072 ± 0.142
41–50	1.084 ± 0.121	1.063	1.056 ± 0.134	1.055 ± 0.128	0.981 ± 0.212	0.941 ± 0.124	1.051 ± 0.129	1.058 ± 0.134
51–60	0.939 ± 0.139	1.024	0.993 ± 0.141	0.932 ± 0.153	1.098 ± 0.174	0.952 ± 0.114	1.053 ± 0.143	1.044 ± 0.132
61–65	0.944 ± 0.128	–	0.952 ± 0.142	0.846 ± 0.145	1.128 ± 0.223	0.970 ± 0.127	1.070 ± 0.142	1.018 ± 0.165
66–70	0.933 ± 0.172	–			1.087 ± 0.201			
71–75	0.878 ± 0.123	–	0.902 ± 0.167	0.826 ± 0.156	1.198 ± 0.261	0.947 ± 0.150	1.068 ± 0.177	0.995 ± 0.182
76–80	0.990 ± 0.208	–			1.143 ± 0.232			
81+	0.890 ± 0.124	–	0.932 ± 0.141	0.871 ± 0.216	1.104 ± 0.162	1.030 ± 0.034^f^	1.093 ± 0.208	1.020 ± 0.184
YAM	1.093 ± 0.139				1.049 ± 0.119			
	**Whole Body**							
21–30	1.193 ± .093	–	1.100 ± 0.079^b^	–	1.224 ± 0.112	–	1.191 ± 0.099^b^	–
31–40	1.167 ± 0.126	–	1.118 ± 0.087	–	1.198 ± 0.111	–	1.202 ± 0.103	–
41–50	1.132 ± 0.116	–	1.119 ± 0.093	–	1.148 ± 0.140	–	1.195 ± 0.106	–
51–60	1.004 ± 0.094	–	1.094 ± 0.098	–	1.140 ± 0.130	–	1.181 ± 0.108	–
61–65	1.054 ± 0.096	–	1.059 ± 0.101	–	1.152 ± 0.119	–	1.164 ± 0.110	–
66–70	1.058 ± 0.140	–	1.033 ± 0.102	–	1.175 ± 0.121	–	1.155 ± 0.111	–
71–75	1.069 ± 0.072	–	1.006 ± 0.102	–	1.219 ± 0.135	–	1.144 ± 0.111	–
76–80	1.120 ± 0.184	–	0.977 ± 0.102	–	1.226 ± 0.121	–	1.128 ± 0.111	–
81+	1.063 ± 0.109	–	0.937 ± 0.102	–	1.186 ± 0.075	–	1.104 ± 0.111	–
YAM	1.201 ± 0.115				1.219 ± 0.119			

Data presented in mean ± standard deviation

BMD values are in g/cm^2^

^a^: Data obtained from Kelly et al. [25]; ^b^: Data obtained from Looker et al. [24]; ^c^: Data obtained from Zhang et al. [20]; ^d^: Data obtained from Goh et al. [13] (data converted from Lunar DXA System to Hologic System using formula provided by Hologic [26]); ^e^: Data obtained from Thoo et al. [27]; ^f^: *n* = 2

Table 4 compares the young adult mean LS BMD of our study (SG-BMD) with other countries, and our prevalence of osteoporosis and osteopenia based on BMD cut-offs derived from our YAM and of other countries. For YAM LS BMD, SG-BMD has the highest LS YAM BMD for women compared to all other countries, with percentage difference range between 0.18–9.99%. In contrast, for men - SG-BMD has the lowest LS YAM BMD score compared to all other countries, with percentage difference between 2.3–10.6%. Prevalence of osteoporosis and osteopenia varied greatly when using different YAM values (Table 4).

**Table 4:** Comparison of young adult mean of SG-BMD vs Other Countries, and prevalence of osteoporosis and osteopenia of Singaporeans using various cut-off values of lumbar spine

	Age Group	Women		Men	
		YAM_**BMD**_	% Difference	YAM_**BMD**_	% Difference
**Singapore**	21–39	1.09 ± 0.14	–	1.05 ± 0.12	–
**NHANES** [24]	20–29	1.06 ± 0.11	2.73	1.06 ± 0.11	0.76
**China** [20]	30–39	1.09 ± 0.13	0.27	1.07 ± 0.14	2.29
**Prevalence of osteoporosis using cut-off values of**		**Prevalence (%)**		**Prevalence (%)**	
**Singapore**		9.3	–	0.7	–
**Hologic**		17.3	60.5	5.6	155.6
**NHANES**		17.3	60.5	5.6	155.6
**China**		18.9	68.1	5.6	155.6
**Prevalence of osteopenia using cut-off values of**					
**Singapore**		48.8	–	23.1	–
**Hologic**		46.3	5.3	19.6	16.4
**NHANES**		40.7	18.1	18.9	20.0
**China**		51.0	4.4	23.1	0.0

Data presented in mean ± standard deviation, and in percentage

Prevalence of osteoporosis and osteopenia are calculated from post-menopausal women and > 50 yr men

% Difference is the percentage of difference between SG-BMD and other countries

There were significant differences between mean T-score of the LS in women using various cut-off values (T_SG_: − 1.13 ± 1.06; T_DXA_: − 1.34 ± 1.33, *p* < 0.001; T_NHANES_: − 1.22 ± 1.39, *p* < 0.001; T_CHN_: − 1.51 ± 1.23, *p* < 0.001). In men, there were significant differences between T_SG_ with T-scores from Hologic, China, and NHANES cut-off values (T_SG_: 0.20 ± 1.53; T_DXA_: 0.33 ± 1.93, p < 0.001; T_NHANES_: 0.53 ± 1.98, p < 0.001; T_CHN_: 0.03 ± 1.79, p < 0.001).

The prevalence of osteoporosis, calculated using LS BMD, was 9.3% in postmenopausal women and 0.7% in men above 50 year of age using T_SG_. However, the prevalence was nearly twice at 17.3% in postmenopausal women, and 8-times greater at 5.6% in men above 50 years of age when using T_DXA_ (Table 4).

Cohen’s weighted kappa (κ_ω_) statistics were presented in Table 5. The main disagreement occurred in the osteopenic group. Among the 28 women identified with osteoporosis by T_DXA_, 46% (*n* = 13) were identified as osteopenia using T_SG_. In men, of the 8 identified as osteoporotic by T_DXA_, 88% (*n* = 7) were identified as osteopenic using T_SG_. The level of agreement between T_DXA_ and T_SG_ is moderate to strong in both postmenopausal women and men aged 50 yr and above (Women: κ_ω_ = 0.81, 95% Confidence Interval (CI): 0.74–0.88; Men: κ_ω_ = 0.85, 95% CI: 0.78–0.93).

**Table 5:** Agreement in identification of lumbar spine osteoporosis between Hologic and Singapore cut-off values

Identification based on Singapore	Identification based on Hologic		
	Normal	Osteopenia	Osteoporosis
**Women**			
**Normal**	59 (86.8)	9 (13.2)	0
**Osteopenia**	0	66 (88.0)	13 (16.5)
**Osteoporosis**	0	0	15 (100.0)
**Men**			
**Normal**	107 (98.2)	2 (1.8)	0
**Osteopenia**	0	26 (78.8)	7 (21.2)
**Osteoporosis**	0	0	1 (100.0)

Values are shown as number of individuals in each subgroup, and percentage of row-wise total. Men: κ_ω_ = 0.85 (95% CI: 0.78–0.93); Women: κ_ω_ = 0.81 (95% CI: 0.74–0.88)

### Post-hoc analysis

Variations of WB and LS BMD show a trend of increase across age groups in men, which was different when compared to other studies [17, 20, 25, 28]. In post-hoc analysis, significant difference was found between age groups in LS BMD as determined by one-way ANOVA (F(8, 181) = 2.256, *p* < 0.05). Post-hoc Tukey’s honestly significant difference (HSD) test showed no difference between all age group in both WB and LS BMD, except between 41 and 50 and 75–80 age group in the LS BMD (*p* = 0.015).

## Discussion

Our results showed consistent differences between the prevalence of osteoporosis and osteopenia when using the reference data provided by the densitometry manufacturer and our derived reference data. With higher reference values, the densitometry provided reference data has the tendency to over-estimate osteoporosis in Singapore’s population. It is well established that measured BMD differs between ethnicities [29]. Using our reference values, the prevalence of osteoporosis was found to be 9.3 and 0.7% for postmenopausal women and men above 50 years of age, respectively. Comparatively, the prevalence of osteoporosis is estimated to be about 2–10 times higher at 17.3, 17.3, and 18.9% for women and 5.6, 5.6, and 5.6% for men, when using reference data from Hologic, NHANES, and China, respectively. Comparison between osteoporosis prevalence derived using reference data from our study, Hologic, and other countries, the percentage differences range between 60.5–155.6%.

A similar discrepancy was reported in another study in South East Asia. In a study of 653 Vietnamese men and women, the prevalence of osteoporosis was 29% in women and 10% in men, with the local reference values. However, the prevalence was much higher - 44% in women and 30% in men, when using the DXA-provided reference values [15]. In a large Chinese study, the young adult BMD reference value was found to be 4–5% lower compared to the US reference values [19]. This resulted in an artificial 2-fold increase in the estimated prevalence of osteoporosis - from 13.3 to 26.7% in women, and from 5.9 to 11.1% in men [19].

The LS BMD of women from this study is higher in all age group, except for the 50–59 age group, when compared to Goh et al. [13]. Furthermore, Goh [13] reported peak LS BMD occurring at 40–49 age group compared to the 31–40 age group in our study. The difference is likely due to cohort effect, for the previous study was conducted about 16 years ago. In this study, a steep BMD decline occurs in women aged 41–50 for both LS and WB BMD. This is best explained by the decrease in oestrogen at menopause, as the onset of menopause occurs between 36 and 59 years of age in Singaporean women [30]. Similar trends in LS BMD of women - with the decline of BMD in the same age range, were reported in studies of other ethnic groups [15, 19, 21]. Our study showed that Singaporean women have higher LS BMD in the younger age group (21-50 yrs) compared to the Caucasians. This may partly be attributed to an earlier onset of menarche in Singaporean women (12 years of age) compared to the Caucasians (13 years of age) [31, 32]. The YAM value for lumbar spine in women was similar to the NHANES and China values. However, the different SD values explained the difference in prevalence. As the WHO definition of diagnosis of osteoporosis depends on the SD value, a small change in SD value may cause notable variation in T-score, resulting in a significant effect on the number of individuals meeting the WHO diagnosis criteria [20]. From the regression model, the relationship between BMD and age in women was best fitted with a polynomial equation of the third degree (Fig. 1), which is consistent with other studies [15, 19]. According to the functional relationship, our data shows Singaporean women reaches predicted peak LS BMD at 31 years old - close to Caucasian (30–39 years old) [24, 33] and Chinese women (34 years old) [19].

Age group at which peak LS BMD occurs in men was similar to the previous study, however, the peak BMD value was higher in our study (1.006 ± 0.115 g/cm^2^ vs 1.049 ± 0.119 g/cm^2^) [27]. LS BMD across all age group was also found to be lower compared to this study [27]. These differences may be due to better nutrition of the current cohort effect compared to the previous cohort from 18 years ago. The lower peak BMD reported, correspond with the low BMD in the current study 31–40 age group (Table 3). The WB BMD and LS BMD of men in our study do not share a similar trend as the women. There is a steep decline in the 31–40 age group, and then an increasing trend in older age groups. Similar trends had been reported for WB [17] and LS BMD [24] elsewhere. Singaporean men have a lower LS BMD in the 21–50 years age group but higher in older age groups compared to the Caucasians. Compared to Caucasians, Singaporean men aged 66 years and older have a higher WB BMD. The relationship between LS BMD with age was best fitted with a positive linear regression equation, and the relationship between WB BMD and age was best fitted with a polynomial equation of the second degree (Fig. 1). Despite the apparent increasing trend in BMD in the older age group, post-hoc analysis revealed no significant difference in BMD within age groups. A similar trend was also reported in the NHANES studies, regardless of race and ethnicity [24]. YAM was found to be lower in Singapore men compared to NHANES and China. Sedentary lifestyle (78%) in Singaporean adolescent may explain the lower YAM BMD [34]. The relationship between age and BMD in older men could be confounded by cohort effect, with the older generation having a healthier lifestyle [35]. However, such a relationship has been reported in other studies [20, 36, 37]. For older men, BMD has been found to increase with age [38]. The apparent increase in BMD have been attributed to degenerative-changes of the LS, such as osteophytes, aortic calcification, vertebral compression fracture, scoliosis, and osteoarthritis [39]. The difference in the rate of spinal BMD diminution was found to be 1 and 10% per decade in men and women, respectively [40]. However, other studies reported that lumbar spine BMD increases at a rate of 1.5–3.5% per decade in men over 60 years old [41–43]. The compound effect of older men physiology and the cohort effect likely explained the lack of significant change in BMD across age in our study.

Ethnicity has been established as a determinant of BMD and the risk of osteoporosis [29]. While the majority of BMD research has compared the local reference values to the Caucasians, it is important to note that variance in BMD values was also reported among different ethnic groups in Asia. For example, the age-specific BMD in Chinese was reported to be lower compared to the Japanese and Koreans [20]. Differences were also found among the different ethnic groups in a country based on geographical locations, possibly due to differences in diet, lifestyle and body size [20]. As a country in South East Asia, while predominantly ethnic Chinese, diet, lifestyle and culture of Singaporean differ from those of people groups in China. Additionally, there are also ethnic variations within Singapore. Singaporean Chinese women have a 40% higher hip fracture rates compared to local Malay and 90% higher than Indian women [9]. Post-hoc secondary analysis of covariance our data showed that there were no significant differences in LS BMD among the three major ethnic group for both men (*p* = 0.692) and women (*p* = 0.802). This result is similar to a previous study [13]. However, significant differences was reported in femoral neck BMD among Singapore women [13]. Though the BMD differences between different ethnicity in Singapore has not been investigated, various influencing risk factors for low BMD, such as obesity, vitamin D deficiency, smoking, alcohol consumption and genetic heterogeneity, have been studied. Ethnic-specific genetic variants and risk factors associated with low BMD warrant future research [9].

Lifestyle is also an established determinant of BMD. As this is a cross-sectional study, inter-generation lifestyle differences may have a cohort effect on the mean BMD of each age group. Singapore’s economic development is unique. Over three decades, it has progressed from a labour-intensive industry to a predominantly modern-day service industry. In 2016, 36.5% of Singaporean adults are reported to be physically inactive, and 69.7% adolescent boys and 83.1% adolescent girls between 11 and 17 years of age are physically inactive [44]. It has been reported that a 10% increase in peak bone mass in children will reduce the risk of osteoporotic fracture by 50% during adult life [45]. Low physical activity in adolescents may increase the risk of low bone mass and osteoporosis in middle to older ages.

To our knowledge, this is the first prevalence study of osteoporotic conditions in Singapore using population-based BMD reference values. While study sample size may not be large, it is randomly selected a priori from a nationally and ethnically representative residential population. Therefore, this population-based dataset adds to the much needed local and South East Asian BMD reference database. Individuals with clinical conditions deemed to interfere with bone metabolism were also excluded from the data analysis. Ideally, peak bone mineral density should be estimated from a longitudinal study, following up participants from the age of 5 to 40, but such a study is not feasible. The DXA lumbar spine BMD was extracted from the whole-body scan, which may affect the accuracy of the BMD obtained. An important limitation is that other skeletal sites, such as femoral neck, Ward’s triangle, trochanter, and total hip, were not available. The study was part of a larger study on body composition and physical performance and so only a whole-body DXA scan using standard protocol was performed (where the regions of interest are head, arm, forearm, leg, ribs, thoracic spine, lumbar spine and pelvis). Site-specific scans of the femoral neck and the hip region were not performed. Another limitation is the exclusion of subjects with fractures or surgeries that affected physical function. Fractures at the hip or femoral neck are known to be more devastating and are important osteoporosis signs. Therefore, further local population-based study on these areas will be needed.

## Conclusion

Our study showed that the prevalence of osteoporotic conditions of Singaporeans is not accurately represented when using reference data provided by the DXA manufacturer; and contributes to local and Asian population reference databases. There could have been an overestimation of osteoporosis and osteopenia by using manufacturer-provided reference data, that may possibly lead to over-treatment in a certain segment of our population. There is an urgent need to establish a South-East Asian BMD reference database for lumbar spine and femur neck to provide an accurate picture of the prevalence of osteoporotic conditions of the population in this region. It is also important to better understand the determinants of BMD and bone health in Southeast Asia.

## Data Availability

The data that support the findings of this study are available from the corresponding author SLW, upon reasonable request. The data are not publicly available due to their containing information that could compromise the privacy of research participants.

## References

[1] Consensus Development Conference (1993). Diagnosis, Prophylaxis, and Treatment of Osteoporosis. Am J Med.

[2] World Health O. WHO Scientific Group on the Assessment of Osteoporosis At Primary Health. World Health 2007(May 2004):1–13.

[3] Johnell O, Kanis J (2005). Epidemiology of osteoporotic fractures. Osteoporos Int.

[4] Morgan SL, Prater GL (2017). Quality in dual-energy X-ray absorptiometry scans. Bone..

[5] Thu WPP, Logan SJS, Cauley JA, Kramer MS, Yong EL (2019). Ethnic differences in bone mineral density among midlife women in a multi-ethnic Southeast Asian cohort. Arch Osteoporos.

[6] Department of Statistics, Singapore (2019). Population Trends.

[7] National P, Talent D (2016). Older Singaporeans to double by 2030.

[8] Koh LKH, Saw S, Lee JJM, Leong K, Lee J, Working N (2001). International Original Article Hip Fracture Incidence Rates in Singapore 1991–1998. Osteoporos Int.

[9] Yong EL, Ganesan G, Kramer MS, Logan S, Lau TC, Cauley JA (2019). Hip fractures in Singapore: ethnic differences and temporal trends in the new millennium. Osteoporos Int.

[10] Chandran M, Lau TC, Gagnon-Arpin I, Dobrescu A, Li W, Leung MYM (2019). The health and economic burden of osteoporotic fractures in Singapore and the potential impact of increasing treatment rates through more pharmacological options. Arch Osteoporos.

[11] Ng CS, Lau TC, Ko Y (2017). Cost of osteoporotic fractures in Singapore. Value Health Regional Issues.

[12] Mithal A, Kaur P (2012). Osteoporosis in asia: a call to action. Current Osteoporosis Reports.

[13] Goh JCH, Low SL, DasDe S (2004). Bone mineral density and hip axis length in Singapore's multiracial population. J Clin Densitom.

[14] Donovan Walker M, Babbar R, Opotowsky AR, Rohira A, Nabizadeh F, Della Badia M (2006). A referent bone mineral density database for Chinese American women. Osteoporos Int.

[15] Ho-Pham LT, T Nguyen UD, Pham HN, Nguyen ND, Nguyen TV, Nguyen UDT (2011). Reference ranges for bone mineral density and prevalence of osteoporosis in Vietnamese men and women. BMC Musculoskelet Disord.

[16] Høiberg M, Nielsen TL, Wraae K, Abrahamsen B, Hagen C, Andersen M (2007). Population-based reference values for bone mineral density in young men. Osteoporos Int.

[17] Lin YC, Pan WH (2011). Bone mineral density in adults in Taiwan: results of the nutrition and health survey in Taiwan 2005-2008 (NAHSIT 2005-2008). Asia Pac J Clin Nutr.

[18] Park EJ, Joo IW, Jang MJ, Kim YT, Oh K, Oh HJ (2014). Prevalence of osteoporosis in the Korean population based on Korea National Health and nutrition examination survey (KNHANES), 2008-2011. Yonsei Med J.

[19] Cheng XG, Yang DZ, Zhou Q, Zhuo TJ, Zhang HC, Xiang J (2007). Age-related bone mineral density, bone loss rate, prevalence of osteoporosis, and reference database of women at multiple centers in China. J Clin Densitom.

[20] Zhang ZQ, Ho SC, Chen ZQ, Zhang CX, Chen YM (2014). Reference values of bone mineral density and prevalence of osteoporosis in Chinese adults. Osteoporos Int.

[21] Iki M, Kagamimori S, Kagawa Y, Matsuzaki T, Yoneshima H, Marumo F (2001). Bone mineral density of the spine, hip and distal forearm in representative samples of the Japanese female population: Japanese population-based osteoporosis (JPOS) study. Osteoporos Int.

[22] Iki M, Tamaki J, Sato Y, Morita A, Ikeda Y, Kajita E (2015). Cohort profile: the Japanese population-based osteoporosis (JPOS) cohort study. Int J Epidemiol.

[23] Hogg R, Tanis E, Zimmerman D (2015). Probability and statistical inference.

[24] Looker AC, Borrud LG, Lumbar Spine HJP (2012). Proximal Femur Bone Mineral Density , Bone Mineral Content , and Bone Area : United States, 2005–2008. Vital Health Stat.

[25] Kelly TL, Wilson KE, Heymsfield SB (2009). Dual energy X-ray absorptiometry body composition reference values from NHANES. PLoS One.

[26] Wilson KE, Hologic Inc (2011). Practical Considerations When Replacing a DXA system.

[27] Thoo FL, Chng SM, Lam KS, Lee JBI, Tan MC, Teh HS (2002). To establish the normal bone mineral density reference database for the Singapore male. Ann Acad Med Singap.

[28] Lynn HS, Lau EMC, Au B, Leung PC (2005). Bone mineral density reference norms for Hong Kong Chinese. Osteoporos Int.

[29] Nam HS, Shin MH, Zmuda JM, Leung PC, Barrett-Connor E, Orwoll ES (2010). Race/ethnic differences in bone mineral densities in older men. Osteoporos Int.

[30] Loh FH, Khin LW, Saw SM, Lee JJM, Gu K (2005). The age of menopause and the menopause transition in a multiracial population: a nation-wide Singapore study. Maturitas..

[31] Chumlea WC, Schubert CM, Roche AF, Kulin HE, Lee PA, Himes JH (2003). Age at menarche and racial comparisons in US girls. Pediatrics..

[32] Agarwal A, Venkat A (2009). Questionnaire study on menstrual disorders in adolescent girls in Singapore. J Pediatr Adolesc Gynecol.

[33] Mazess RB, Barden H (1999). Bone density of the spine and femur in adult white females. Calcif Tissue Int.

[34] Guthold R, Stevens GA, Riley LM, Bull FC (2020). Global trends in insufficient physical activity among adolescents: a pooled analysis of 298 population-based surveys with 1·6 million participants. Lancet Child Adolesc Health.

[35] Warming L, Hassager C, Christiansen C (2002). Changes in bone mineral density with age in men and women: a longitudinal study. Osteoporos Int.

[36] Chanchairujira K, Chung CB, Kim JY, Papakonstantinou O, Lee MH, Clopton P (2004). Intervertebral disk calcification of the spine in an elderly population: radiographic prevalence, location, and distribution and correlation with spinal degeneration. Radiology..

[37] Looker AC, Melton LJ, Borrud LG, Shepherd JA (2012). Lumbar spine bone mineral density in US adults: demographic patterns and relationship with femur neck skeletal status. Osteoporos Int.

[38] Henry MJ, Pasco JA, Korn S, Gibson JE, Kotowicz MA, Nicholson GC (2010). Bone mineral density reference ranges for Australian men: Geelong osteoporosis study. Osteoporos Int.

[39] Anderson KB, Holloway-Kew KL, Mohebbi M, Kotowicz MA, Hans D, Pasco JA (2018). Is trabecular bone score less affected by degenerative-changes at the spine than lumbar spine BMD?. Arch Osteoporos.

[40] Mazess RB, Barden HS, Drinka PJ, Bauwens SF, Orwoll ES, Bell NH (1990). Influence of age and body weight on spine and femur bone mineral density in U.S. white men. J Bone Miner Res.

[41] Cauley JA, Fullman RL, Stone KL, Zmuda JM, Bauer DC, Barrett-Connor E (2005). Factors associated with the lumbar spine and proximal femur bone mineral density in older men. Osteoporos Int.

[42] Dennison E, Eastell R, Fall CHD, Kellingray S, Wood PJ, Cooper C (1999). Determinants of bone loss in elderly men and women: a prospective population-based study. Osteoporos Int.

[43] Yoshimura N, Kinoshita H, Danjoh S, Takijiri T, Morioka S, Kasamatsu T (2002). Bone loss at the lumbar spine and the proximal femur in a rural Japanese community, 1990-2000: the Miyama study. Osteoporos Int.

[44] Guthold R, Stevens GA, Riley LM, Bull FC (2018). Worldwide trends in insufficient physical activity from 2001 to 2016: a pooled analysis of 358 population-based surveys with 1·9 million participants. Lancet Glob Health.

[45] Bonjour JP, Chevalley T, Ferrari S, Rizzoli R (2009). The importance and relevance of peak bone mass in the prevalence of osteoporosis. Salud Publica Mex.

